# Long Term Recidivism Rates Among Individuals at High Risk to Sexually Reoffend

**DOI:** 10.1177/10790632221139166

**Published:** 2022-11-16

**Authors:** R. Karl Hanson, Seung C. Lee, David Thornton

**Affiliations:** 1 Department of Psychology, 6339 Carleton University, Ottawa, ON, Canada; 2 SAARNA: Society for the Advancement of Actuarial Risk Need Assessment, ON, Canada; 3 FAsTR LLC, Madison, WI, USA

**Keywords:** sexual recidivism, risk assessment, Static-99R, Static-2002R

## Abstract

Preventive detention provisions in the US and Canada assume we can identify, in advance, individuals at high risk for sexual recidivism. To test this assumption, 377 adult males with a history of sexual offending were followed for 20 years using Canadian national criminal history records and Internet searches. Using previously collected information, a high risk/high need (HRHN) subgroup was identified based on an unusually high levels of criminogenic needs (*n* = 190, average age of 38 years; 83% White, 13% Indigenous, 4% other). A *well above average* subgroup of 99 individuals was then identified based on high Static-99R (6+) and Static-2002R (7+) scores. In the HRHN group, 40% reoffended sexually. STATIC HRHN norms overestimated sexual recidivism at 5 years (Static-99R, *E/O* = 1.44; Static-2002R, *E/O* = 1.72) but were well calibrated for longer follow-up periods (20 years: Static-99R, *E/0* = 1.00; Static-2002R, *E/O* = 1.16). The overall sexual recidivism rate for the *well above average* subgroup was 52.1% after 20 years, and 74.3% for any violent recidivism. The highest risk individuals (top 1%) had rates in the 60%–70% range. We conclude that some individuals present a high risk for sexual recidivism, and can be identified using currently available methods.

## Introduction

Individuals deemed high risk to sexually reoffend can be subject to exceptional legal measures, such as the Sexually Violent Person (SVP) provisions in many US states (Prentky et al., 2015) and the Dangerous Offender (DO) designation in Canada (Criminal Code of Canada, 1985). These provisions allow for indefinite detention, with release predicated on reassessments indicating the individual’s risk has declined below some acceptable threshold. Psychologists who provide expert opinions in such cases often rely on specialized sexual recidivism risk tools (Kelley et al., 2020).

The definition of high likelihood to reoffend varies across settings, and may not be explicitly defined. SVP laws in the USA commonly use the term “likely” in their statutes, which, in some states, has been clarified to mean “more likely than not” either in statute or case law (Knighton et al., 2014). This threshold implies absolute rates of greater than 50%. Other states do not define “likely”, and still other states use phrases such as “highly probable”, which implies a rate greater than “more likely than not”. Furthermore, some SVP laws specifically exclude quantitative interpretation. An additional complication is that the likelihoods stipulated by SVP laws are usually understood as referring to lifetime risk, which may not align with the information provided by most actuarial tables (i.e., the recidivism rate estimates associated with the scores of risk tools). Actuarial tables can be biased upwards because they include sexual crimes that do not meet the statutory definition of sexual violence (e.g., exhibitionism, possession of child abuse images). On the other hand, the rates can be biased downward because they only include *detected* offences within a discrete follow-up period (e.g., 5 years, 10 years). In practice, decision-makers routinely interpret expected rates somewhat lower than 50% as meeting the “more likely than not” threshold.

In Canada, the Dangerous Offender (DO) designation allows for the preventive detention of individuals deemed high risk for committing a serious personal injury offence. Although the outcome of concern includes both sexual and nonsexual violence, two-thirds of individuals with DO designations have an index sexual offence (Public Safety Canada, 2020). The threshold for high risk is that “the trial judge must be satisfied beyond a reasonable doubt that there is a “likelihood” that the offender will [commit another serious injury offence]” (R. v Currie, 1997, paragraph 42, quotation marks in the original). To our knowledge, this likelihood has never been given an explicit quantitative interpretation. In practice, however, the DO designation is restricted to a very small proportion of the highest risk individuals (Blais & Bonta, 2015). In 2017–2018, there were close to 90,000 cases involving crimes against persons, of which less than 50 resulted in a DO designation (Public Safety Canada, 2020) - a rate of less than 1 in 1500.

Decisions about the likelihood of any future outcome should be informed by base rates (Kahneman et al., 2021). Previous reviews have observed sexual recidivism rates of between 5% and 15% after 5 years, increasing to 10%–20% when the follow-up is extended to 10 years (Hanson & Bussière, 1998; Harris & Hanson, 2004; Helmus et al., 2012a). In recent routine samples, sexual recidivism rates of 2%–8% are common (Alper & Durose, 2019; Boccaccini et al., 2017; Helmus et al., 2021a). Helmus et al., (2021a), for example, observed an average sexual recidivism rate of 4.2% among 13 studies published between 2013 and 2021 (combined sample of 48,025).

Not all individuals with a history of sexual offending are equally likely to reoffend. Rates vary based on well-established risk factors (see meta-analyses by Hanson & Bussière, 1998; Hanson & Morton-Bourgon, 2009; Mann et al., 2010). These risk factors can be divided into static, historical variables (e.g., prior sexual offences, any unrelated victims) and indicators of psychological and community adjustment that can be used to guide supervision and treatment (i.e., dynamic risk factors or criminogenic needs, such as sexual preoccupation and negative attitudes towards supervision). Although static and dynamic risk factors are based on different information, both types of variables predict recidivism because they are markers for enduring, risk relevant propensities (Mann et al., 2010).

Given the relatively low overall base rate, it is not clear what the upper end of the recidivism rates should be for individuals who have multiple, severe risk relevant propensities. Although it is easy to retrospectively identify individuals with very high recidivism rates (i.e., by post hoc analyses), recidivism rate estimates only have practical utility when they can be specified in advance. Consequently, the confidence with which evaluators can make high risk determinations depends on the *calibration* of their risk assessments (i.e., correspondence between the expected rates and the observed rates in replication studies; see Helmus & Babchishin, 2017; Hanson, 2022, Part IV).

The highest rates estimated by sexual recidivism risk tools are 60%–80% (Helmus et al., 2021b; Olver et al., 2020). The Violence Risk Scale – Sexual Offense version (VRS-SO; Olver et al., 2007), for example, estimates the 10-year sexual recidivism rate to be 80% for individuals with the maximum score (72) and no treatment gains (Olver et al., 2021). It is unclear, however, whether there are real cases with such extreme values. The highest VRS-SO score in the workbook (Olver et al., 2020) is 67, and there were only 10 cases out of 913 that had scores of 57 or higher. According to the VRS-SO, the top 1% (scores 57 or higher) would be expected to have 10-year sexual recidivism rates of 50%–60%.

Static-99R and Static-2002R (Helmus et al., 2012b) are the most commonly used sexual recidivism risk tools, with routine use in the US (Kelley et al., 2020), Canada (Bourgon et al., 2018), Australia, New Zealand, Europe, and East Asia (Helmus et al., 2022; Neal & Grisso, 2014). As their names imply, they privilege static risk factors, such as age and criminal history. They are considered actuarial risk tools because they use an explicit method of combining the items into total scores, and these scores are associated with expected recidivism rates (Helmus et al., 2021b). The user guidance for Static-99R and Static-2002R present sexual recidivism rates for three follow-up periods (5, 10, and 20 years) and for two reference groups: a) routine/complete samples (Lee & Hanson, 2021) and b) samples preselected to be high risk/high need (HRHN; Hanson et al., 2016; Helmus et al., 2021b). The need for separate tables was motivated by higher-than-expected sexual recidivism rates in samples that were preselected as high risk. Examples of the high risk/high need samples include individuals referred to high intensity treatment programs, or for assessments to determine whether they qualify as Dangerous Offenders (based on Canadian criminal law). According to user guidance, HRHN samples are conceptually distinguished from routine samples based on an unusually high density of dynamic risk factors/criminogenic needs (Hanson et al., 2016). The major differences between the sexual recidivism rates for the routine/complete and HRHN samples are for the lower risk and average risk levels; in these ranges, the recidivism rates are materially higher for the HRHN groups. In contrast, the expected recidivism rates associated with the highest scores are similar for both reference groups. The highest named risk level for Static-99R and Static-2002R is Level IVb – *well above average*, representing the top 10%, for which the expected sexual recidivism rates are 25%–45% after 5 years, and 45%–65% after 20 years (Helmus et al., 2021b).

There are several significant qualifications to STATIC (Static-99R and Static-2002R) expected recidivism rate tables. First, the HRHN norms were based on a limited number of studies (*k* = 5 for Static-99R; *k* = 2 for Static-2002R). Second, the 20-year rates are not observed values; instead, they are projected from the observed rates at 5-year and 10-year follow-up periods using the procedure described in Thornton et al. (2021). A further limitation is that there is considerable variability in the recidivism base rates across studies for reasons not fully understood. Recent calibration studies of the 2016 Static-99R recidivism norms (Baudin et al., 2021; Boccaccini et al., 2017; Helmus et al., 2021a; Lee et al., 2016, 2018), have found adequate to poor calibration; the general pattern is that new samples display lower recidivism rates than suggested by the 2016 norms (e.g., average *E/O* index of 1.88 in five field validity studies; Helmus et al., 2021a). In response to such findings, the routine/complete norms for Static-99R have been revised (Lee & Hanson, 2021). There is insufficient research, however, to determine whether the 2016 HRHN norms similarly need updating.

The STATIC 20-year estimates in particular could benefit from calibration studies. In some contexts, the relevant question is the lifetime risk of sexual offending. Given that long term studies are difficult (and always dated), a reliable method of extrapolating from shorter follow-up periods would be of considerable utility. Thornton et al., (2021) have proposed a generic mathematical model that can be used to translate observed sexual recidivism rates at any specific follow-up period (e.g., 5-years) to rates at any other follow-up period (e.g., 1-year, 15-years). Thornton’s model is based on a predictable and consistent decline in sexual recidivism rates the longer that individuals remain sexual offence free in the community (Hanson et al., 2018). In the model, the 20-year rates are treated as equivalent to the lifetime rates. Residual risk after 20 years is not zero; however, it is so small as to be indistinguishable from the rate of spontaneous out-of-the-blue sexual offences in the general male population. Although general time free effects have been replicated (e.g., Moore, 2018), the specific 20-year projections for Static-99R and Static-2002R have not.

## Overview of Current Study

A primary goal was to estimate the upper range of sexual recidivism rates that could be specified in advance. Given that the expected rates were based on norms for Static-99R and Static-2002R risk tools, this research question could also be stated as testing the calibration of these measures. Twenty-year estimates for the high risk/high need (HRHN) reference group (Helmus et al., 2021b) for the highest STATIC scores (95^th^ percentile) are in the 50%–65% range, which is substantially higher than rates typically observed in contemporary follow-up studies (<10%). Finding observed rates over 50%, however, would require exceptionally long follow-up of individuals who were preselected to be high risk for sexual recidivism.

The opportunity to address these research questions was provided by an extended follow-up (median of 20.5 years) of a research sample of approximately 400 men who had committed sexual offences in the 1990s. The original sample was preselected to be above average risk - half had already sexually reoffended while on community supervision (Hanson & Harris, 2000a, 2000b). Using variables collected during the initial research, a subgroup was identified that matched the definition of a high risk/high need sample asserted by the STATIC norms (i.e., unusually high levels of criminogenic needs; Hanson et al., 2016). Static-99R and Static-2002R scores were coded retrospectively based on case file and criminal history information.

### Hypotheses

We expected the predictive accuracy of Static-99R and Static-2002R prediction tools to be acceptable both in terms of discrimination (*AUC* values around .70) and calibration (nonsignificant *E/O* values close to 1). The coders had been trained by certified trainers and used research data that has proven informative in previous studies (Hanson & Harris, 2000b). Furthermore, Static-99R and Static-2002R have performed well in this jurisdiction (Canada; Helmus et al., 2012b).

Given the preselection, we expected observed sexual recidivism rates to be closer to the HRHN norms than to the routine/complete norms. Nevertheless, our confidence was tempered by knowledge that the 2016 Static-99R routine/complete norms had overestimated the observed rates in recent samples (Helmus et al., 2021a). We had more confidence in the calibration for the 5-year and 10-year rates than we did in the 20-year rates because the very long-term estimates require additional assumptions concerning the shape of the recidivism survival function. Although the general shape of the hazard function has been replicated, the accuracy (calibration) of the specific method used by the STATIC measures for estimating the 20-year rates (Thornton et al., 2021) has yet to be empirically tested in a new sample.

## Method

### Subjects

The current sample was selected from men previously identified for Hanson & Harris, 2000a, 2000b study of dynamic (changeable) risk factors for sexual recidivism. The original sample included 208 men known to have sexually reoffended while on community supervision, roughly matched on criminal history and location/jurisdiction to 201 men who were on community supervision for a sexual crime without reoffending. Cases were drawn from Canada’s federal prison service (Correctional Service of Canada [CSC]) and all provincial correctional systems (except for Prince Edward Island). From the original sample of 409, 7.8% (*n* = 32) were excluded due to lack of recidivism information; the sample was further reduced to 190 individuals preselected to be high risk using the criteria described below.

On average, the presumptively high-risk individuals (*n* = 190) were 38 years old (*SD* = 10.8, range from 18 to 78 years) when released into the community between 1986 and 2016 (*Mdn* = 1996). Eighty-three percent (157/190) were White, 13% (*n* = 24) were of Indigenous heritage, 4% were from other ethnocultural groups (e.g., Black, Asian). The average Static-99R and Static-2002R total scores were 5.1 (*SD* = 2.29; range of −3 to 10; *Md*n = 5) and 6.9 (*SD* = 2.31; range of −1 to 11; *Mdn* = 7), respectively.

The recidivism dataset used in this study has been previously used by Aelick et al. (2020) in a study of mental health variables, and by Lee et al.’s (2020) in a meta-analysis on the predictive validity of sexual recidivism risk tools for individuals of Indigenous heritage in Canada.

### Measures

#### Structured Risk Assessment Need Framework

The SRA framework was used to identify a presumptively high risk/high need (HRHN) subgroup from the original sample. Some measure of dynamic risk factors was necessary because the STATIC HRHN norms are intended to apply to individuals who have been preselected to have unusually high levels of criminogenic needs. Step two of the SRA system provides a flexible method of quantifying the intensity and diversity of long-term vulnerabilities for sexual recidivism risk (see Thornton, 2016). The variables available did not allow for reliable scoring of other validated measures of criminogenic needs, such as STABLE-2007 (Brankley et al., 2021) or the VRS-SO (Olver et al., 2007).

The long term vulnerabilities from the SRA system were scored from indicators for each of the four Need Domains postulated by SRA (Sexual Interests, Distorted Attitudes, Relational Style, and Self-Management). For each domain a summary score between 0 and 2 was constructed. These domain summaries were then summed, yielding a Need Score from 0 to 8. See Supplemental Appendix for further details.

For the present purposes, it was also necessary to define a threshold Need score to classify individuals as “high need”. A score of 4.0 or above was used for this purpose. Such a score can be obtained by having serious, multi-need problems within two domains; having a serious single-need problem in all four domains; or some mixture of serious, single and multi-need problems in three domains. It also corresponds to someone halving at least half of the possible criminogenic need points. This is consistent with the level of criminogenic need that is commonly found in existing HRHN samples (see Supplemental Appendix). The score of 4 also approximately divided the present sample into approximately equal sized higher and lower Need groups. In the full sample with sufficient information to scores the SRA (*n* = 367), the average SRA score was 4.0 (*SD* = 1.7, *Mdn* = 4.2, range from zero to 7.7).

Importantly, the author who constructed the Need score and chose the threshold was blind to the recidivism data.

#### Static-99R

Static-99R (Hanson & Thornton, 2000; Helmus et al., 2012b; Thornton, 2002) is an empirical actuarial risk tool designed to assess risk of sexual recidivism among adult men charged or convicted of a sexual motivated offense. It contains 10 items related to demographic information (age, relationship history) and criminal history (e.g., prior sexual offences, prior nonsexual violence, any male victims). Total scores (range from −3 to 12) are calculated by summing all item points and can be used to place individuals in one of five risk levels: Level I – *very low risk* (scores of −3 to −2), Level II – *below average risk* (scores of −1 to 0), Level III- *average risk* (scores of 1–3), Level IVa – *above average risk* (scores of 4–5), and Level IVb – *well above average risk* (scores of 6 or higher; Hanson et al., 2017). Total scores can be interpreted in terms of percentile ranks, relative risk, and absolute recidivism rates for 5-year, 10-year, and 20-year follow-up periods (Helmus et al., 2021b). Meta-analyses based on large samples (>15,000) indicate moderate ability to discriminate between sexual recidivists and nonrecidivists (average AUC values of .66–.68; Helmus et al., 2021a, 2022).

Rater reliability of the Static-99R total scores was not available in the current study because they were calculated from five variables already coded in the dataset (e.g., age, any male victim) and five criminal history items specifically coded for this study. The rater reliability for these five newly coded items ranged from .71 to .98 (median Kappa of .88 based on 59 cases coded by two raters). Previous studies have found overall good interrater reliability of the Static-99R total scores (intraclass correlation coefficient [ICC] ranging from .78 to .96; Gonçalves et al., 2020; Hanson et al., 2014b; McGrath et al., 2012; Raymond et al., 2020; Stephens et al., 2017).

#### Static-2002R

Like Static-99R, Static-2002R (Hanson & Thornton, 2003; Hanson et al., 2010) is an empirical, actuarial risk tool for estimating the likelihood of sexual recidivism among adult males based on commonly available demographic and criminal history information. It was developed to improve conceptual consistency and increase predictive accuracy over Static-99. Subsequent research, however, has found Static-2002R and Static-99R to have similar predictive accuracy, and that each contributes incrementally to the likelihood of sexual recidivism (Babchishin et al., 2012b; Lehmann et al., 2013). Total scores (ranging from −2 to 13) are calculated by summing all item points and can be used to place individuals in one of five risk levels: Level I – *very low risk* (scores of −2 to −1), Level II – *below average risk* (scores of 0–1), Level III – *average risk* (scores of 2–4), Level IVa – *above average risk* (scores of 5–6), and Level IVb – *well above average risk* (scores of 7 or higher; Hanson et al., 2017). Total scores can be interpreted in terms of percentile ranks, relative risk, and absolute recidivism rates for 5-year and 20-year follow-up periods (Helmus et al., 2021b).

Rater reliability for Static-2002R total scores was not available because it calculated from variables already coded in the dataset (e.g., age, any male victim) and four items that were specifically coded for this study. The rater reliability for these four newly coded items ranged from .76 to .94 (median Kappa of .89 based on 59 cases coded by two raters). Previous studies have found good interrater reliability for Static-2002/R total scores (ICCs ranging from .87 to .98; Helmus & Hanson, 2007; Jung et al., 2017; Reeves et al., 2018; Smid et al., 2014).

### Identifying Individuals at High Risk for Sexual Recidivism

Two high risk subgroups were constructed from the 409 original cases. The first subgroup was intended to resemble the preselected high risk/high need (HRHN) samples in the STATIC norms. According to Hanson et al. (2016), HRHN samples have high average STATIC scores, and unusually high levels of criminogenic risk factors (i.e., riskiest 20%). In the current sample, the average Static-99R score was in the *above average* range (*M* = 4.3, *SD* = 2.4, *n* = 407). Using a SRA Need score of 4 as the threshold for high levels of criminogenic needs (see above), the HRHN subgroup comprised approximately half of the original sample (200 out of 407 cases; *n* = 190 with recidivism information). The average Static-99R score in the HRHN subgroup was 5.1 (*SD* = 2.3, range from −3–10). The syntax used to create the Need Score and the decision threshold for high risk were made blind to recidivism information.

The second high risk subgroup, the w*ell above average* group, not only had unusually high criminogenic needs, but also unusually high STATIC scores. Unusually high STATIC scores were defined as an average score on the Static-99R and Static-2002R in the well above average range (Level IVb). Given that the raw scores of Static-99R and Static-2002R are in different metrics, these scores were averaged using the metric of logged hazard ratios, consistent with previous studies (Babchishin et al., 2012a, 2012b; Lehmann et al., 2013, see Appendix). Overall, 39.3% (160/407) individuals in the complete sample had average STATIC scores in the well above average range (Level IVb), of which 75% (105/140) also had high SRA scores (4+; SRA scores were not available for 20 of the 160 cases). For the recidivism analyses, the *well above average* group comprised individuals who had complete information on Static-99R, Static-2002R, and SRA, as well as recidivism information (*n* = 99). The average Static-99R score in the *well above average* group was 6.8 (*SD* = 1.1, range from 5 to 10). The average SRA Need score was 5.4 (*SD* = 0.95, range of 4–7.67) in this group. Although high SRA scores and high STATIC scores were intended to be separate criteria, none of the individuals with high STATIC scores had an SRA score lower than four.

### Recidivism

Our primary source of recidivism information was the Canadian Police Information Centre (CPIC) criminal history records held by the Royal Canadian Mounted Police (RCMP). These records are intended to record all convictions across Canada, as well as some charges. In addition, news articles were searched via Google using personal identifiers (e.g., full name plus the province where most prior offences occurred). Forty-two relevant news articles were identified; in all cases, the individuals in the news articles had already been identified as a sexual recidivist based on RCMP records. The offence descriptions in the news reports, however, allowed the researchers to classify six of these events as contact sexual recidivism and two as non-contact sexual recidivism. It is not uncommon that publicly available media informs academic research on serious crime (Laporte et al., 2003; Tanguay, 2004), including the measurement of sexual recidivism (Hanson et al., 2015).

The minimum threshold for recidivism was a charge; however, given the primary source of information used (CPIC), the vast majority of recidivism events were convictions. Out of the initial sample of 409 cases, recidivism information was obtained for 377 (92.2%). Records could be unavailable in 2017 because the individual had been deceased for many years, pardoned, or for unknown reasons (all 409 cases had CPIC records in 1997). The length of follow-up was sufficient (20+ years) that it is likely that some of the individuals with missing records had reoffended prior to their records going missing.

Follow-up time began a) at the date of assessment for the original non-recidivist group, or b) after release from the previously identified recidivism event for the recidivist group in the original study, i.e., the index offences of the recidivist group in the original study were replaced with the date of their previously identified recidivism events, creating new at-risk dates for the recidivist group. Follow-up ended with death (*n* = 34), deportation (*n* = 1), the date of the most recent CPIC record (2017), or the start of a period of incarceration that extended into one of these dates. Mortality was only noted when it appeared in the CPIC or news records (mortality information was not requested from Statistics Canada). The time at-risk prior to sexual recidivism did not include time incarcerated for nonsexual offences after the index offence. Overall, 34 individuals were known to have spent a month or more incarcerated prior to the survival end date for sexual recidivism (median of 4.7 months, range from 1 month to 7.8 years, *n* = 34).

In the full sample with recidivism information (*n* = 377), the average follow-up time was 19.6 years (*Mdn* = 20.5, range from 3 months to 35.6 years). Most of the sample was followed for at least 20 years (65.5%, 247/377); however, few individuals were followed for more than 25 years (3.2%, 12/377). The additional length of follow-up after 20 years was usually only a year or two (mean of 1.8 years, or 21.8 years total). Among the HRHN subgroup, 62% (118/190) were followed for longer than 20 years (*Mdn* = 21.5, range from 3 months to 31 years).

Sexual recidivism included both contact and noncontact sexual crimes. Violent recidivism included nonsexual violent offences (e.g., assault), arson, and contact sexual offences. Classification was based primarily on the name of the offence, although sexual motivation was attributed for certain nonsexual offences when justified by associated information (e.g., news reports, sexual charge followed by a nonsexual violent conviction).

### Procedure

The original data were collected in 1996–1997 by four trained researchers working under the supervision of a project manager employed by the Solicitor General of Canada (now Public Safety Canada; see Hanson & Harris, 2000b). Probation and parole officers throughout Canada were asked to identify individuals who had recently sexually reoffended while on community supervision. These cases were then matched with individuals who had not reoffended. Case information was extracted from file review as well as from structured interviews with the supervising officers.

For the current study, the previously collected case information was linked to recidivism information collected in 2017. Two graduate-level research assistants working for Public Safety Canada coded the recidivism data along with items needed to score risk tools. The 2017–2018 update also resulted in some minor edits and corrections to the original data (e.g., revised at-risk dates).

The current study was conducted under a data sharing agreement with Public Safety Canada. This study received ethics approval from Carleton University Research Ethics Board-B (Project # 115736). All statistics in this manuscript were verified independently by the first and second authors using either Microsoft Excel, IBM/SPSS, or R Statistics.

### Analyses

#### Discrimination

Two statistics were used to describe the extent to which recidivists were different from nonrecidivists (i.e., discrimination): a) the Area Under the Curve (*AUC*), and b) slope coefficients from logistic regression.

##### Area Under the Curve

Area under the curve values range from zero to 1, and express the probability that randomly selected individual who has reoffended has a higher score than a randomly selected nonrecidivist. Following Cohen’s (1988) conventions for Cohen’s *d,* AUC values of .56, .64, and .71 are considered small, moderate, and large, respectively (Rice & Harris, 2005). No association between the predictor and the outcome is indicated by an AUC value of .50.

##### Logistic Regression Slope Coefficients

Slope coefficients indicate the amount of change in the likelihood of the outcome based on a one-unit increase in the risk tool. The coefficients (*B1*) are logged odds ratios (logits), such that exp(*B1*) are odds ratios. An odds ratio of 1.0 (*B1* of zero) indicate no relationship between the predictor and the outcome. Absolute values of the slope coefficients are difficult to interpret because they vary based on the scaling of the risk tool. In previous meta-analyses of preselected high risk/high need samples, the average odds ratio for Static-99R predicting sexual recidivism was 1.28 for 5-years, and 1.26 for 10-years; for Static-2002R, the average odds ratio was 1.24 at 5-years (Hanson et al., 2016, Table 7).

#### Calibration

Two statistics were used to measure the similarity between the observed and expected values (calibration): a) the *E/O* index and b) meta-analysis of logistic regression intercept coefficients (Hanson, 2022). The expected values were from the HRHN recidivism rate tables for Static-99R (5-year, 10-year, 20-year) and Static-2002R (5-year, 20-year; Helmus et al., 2021b). Some STATIC scores do not have associated recidivism rates because they were weakly populated (*n* < 10) in the normative samples. The expected rates for these scores were considered equal to the closest populated value. For example, the High Risk/High Needs norms provide rates for scores of −1 (e.g., 5.6% after 5 years) but not for scores of −2 or −3. Consequently, cases with scores of −2 or −3 were assigned an expected risk level of 5.6% after 5 years, the same as for the score of −1. The expected values for 15 years were calculated for this study using the methods described by Thornton et al. (2021). The observed rates for 5 years and 10 years were calculated using fixed follow-up. The observed rates for 15 years and 20 years were calculated using Kaplan-Meier survival analysis.

##### E/O Index

The *E/O* index is a measure of calibration in which the expected number of recidivists is divided by an observed number of recidivists (Hanson, 2017; Viallon et al., 2009). Perfect calibration is indicated by a value of 1.0. When the observed values were based on fixed follow-up, the 95% confidence intervals were estimates using the following equations (Equations (13.1) and (13.2) from Hanson, 2022; Rockhill et al., 2003):

$$Upper\;Limit\;of\;95\%\;CI\;of\;\frac{E}{O}\;Index=\left(\frac{E}{O}\right)e^{\left(1.96\sqrt{\frac{1}{O}}\right)}$$

$$Lower\;Limit\;of\;95\%\;CI\;of\;\frac{E}{O}\;Index=\left(\frac{E}{O}\right)e^{\left(-1.96\sqrt{\frac{1}{O}}\right)}$$

When the observed values were based on Kaplan-Meier survival analysis, the following equations were used for the 95% confidence intervals (Equations (13.3) and (13.4) from Hanson, 2022; equation (14) in Viallon et al., 2009):

$$Upper\;Limit\;of\;95\%\;CI\;of\;\frac{E}{O}\;Index=\left(\frac{E}{O}\right)e^{\left(1.96\left[\frac{SE}{O}\right]\right)}$$

$$Lower\;Limit\;of\;95\%\;CI\;of\;\frac{E}{O}\;Index=\left(\frac{E}{O}\right)e^{\left(-1.96\left[\frac{SE}{O}\right]\right)}$$

In the above equations, *SE* is the Greenwood approximation of the standard error (equation (10.8) in Singer & Willet, 2003), expressed in the units of cases (not proportions).

##### Comparing Logistic Regression Parameters

Calibration was also examined by comparing the intercept values from logistic regression to those in the HRHN normative samples (Hanson et al., 2016). For both Static-99R and Static-2002R, the intercepts were centered on the median value in routine/complete samples (score of 2 for Static-99R; 3 for Static-2002R); as such, the *B0*’s represents the expected recidivism rates for individuals with a score of 2 (Static-99R) or 3 (Static-2002R), expressed in logit units (ln[p/{1 − p}]). Centering on median values allows direct comparison with the B0’s reported in the STATIC norms.

Differences between the parameters in the current sample and those of the norms were tested using fixed-effect meta-analysis (Borenstein et al., 2021) conducted with the package “metafor” (Version 3.0–2; Viechtbauer, 2010) for the statistical software R (Version 4.0.3; R Core Team, 2020).

## Results

In the full sample with recidivism information, 31.6% (119/377) were known to have committed a new sexual crime during the 20+ year follow-up period. Median follow-up time was 20.5 years and ranged from 3 months (one individual died shortly after release) to 35.6 years. Overall, 94.7% (357) were followed for at least 10 years, 89.1% (336) for at least 15 years, and 65.5% (247) for at least 20 years (all times exclusive of time in custody). The most serious sexual crime was a contact sexual crime in 109 cases and a non-contact sexual crime in 10 cases. There were 16 additional cases who violated their conditions of supervision for presumably sexual motivations (e.g., victim grooming, but no known offences); these cases were not included in the definition of sexual recidivism. In the full sample, 44.0% (166/377) were known to have committed a new violent offence (non-sexual violence, arson, or contact sexual offence) and 54.6% any new criminal offence (206/377). Among the 190 cases identified as High Risk/High Need (HRHN), the recidivism rate for sexual crimes was 40.0% (76/190), 56.3% (107/190) for violent crimes, and 65.5% (131/190) for any crime. Six of the HRHN cases received a Dangerous Offender designation for their recidivism event.

Both Static-99R and Static-2002R showed large relationships with sexual recidivism in the full dataset (*n* = 377, *AUC* = .717, *SE* = .028; the same values for both Static-99R and Static-2002R, ragged follow-up). The average of Static-99R and Static-2002R scores (AUC = .725, SE = .027) was not significantly different from Static-99R (*Z* = 1.11, *p* = .266) or Static-2002R (*Z* = 0.735. *p* = .462; using the Delong test in IBM/SPSS version 27). In the HRHN subsample, all the STATIC measures showed moderate relationships with sexual recidivism (*n* = 190; Static-99R, *AUC* = .671, *SE* = .039; Static-2002R, *AUC* = .685, *SE* = .039; Average of 99R + 02R, *AUC* = .681, *SE* = .039). The raw SRA need scores (without dichotomization) showed a moderate relationship to sexual recidivism in the full data set (*n* = 342, *AUC* = .642, *SE* =.031) and was incremental to the Static-99R (logistic regression incremental *B* of 0.170, *SE* = 0.086, *p* = .048) but not Static-2002R (*B* of 0.146, *SE* = .089, *p* = .102), or the Static-99R + Static-2002R average (*B* = 0.143, *SE* = .089, *p* = .106).

When calibration was examined using the *E/O* index, the observed 5-year sexual recidivism rates for the HRHN subsample were lower than the expected HRHN values for both Static-99R (E/O = 1.44) and Static-2002R (E/O = 1.72; see Table 1). In contrast, the observed 10-year rates for Static-99R were close to the expected HRHN values (E/O = 1.13), as were the 15-year rates (E/O = 0.96) and 20-year rates (E/O = 1.00; see Table 2). Similarly, the observed 15-year and 20-year rates for Static-2002R were close to the expected HRHN values (E/O index of 1.09 and 1.16, respectively; see Table 3).

**Table 1.** Calibration Analyses (E/O Index) with High Risk/High Need Sexual Recidivism Norms for Static-99R and Static-2002R.

		*N*	Expected Recidivism		Observed Recidivism		E/O	95% CI
			*n* of Recidivists (E)	Percent (E)	*n* of Recidivists (O)	Percent (O)		
Static-99R								
5-year								
I	Very low	1	0.056	5.6	0	0		
II	Below average	6	0.42	7.0	0	0		
III	Average	39	4.82	12.4	3	7.7	1.61	0.52, 4.98
IVa	Above average	51	9.95	19.5	7	13.7	1.42	0.68, 2.98
IVb	Well above average	87	26.51	30.5	19	21.8	1.40	0.89, 2.19
All		184	41.76	22.7	29	15.8	**1.44**	**1.001, 2.07**
10-year								
I	Very low	1	0.11	10.6	0	0		
II	Below average	6	0.76	12.6	0	0		
III	Average	39	8.03	20.6	5	12.8	1.61	0.67, 3.86
IVa	Above average	49	14.72	30.0	10	20.4	1.47	0.79, 2.74
IVb	Well above average	87	36.46	41.9	38	43.7	0.96	0.70, 1.32
All		182	60.07	33.0	53	29.1	1.13	0.87, 1.48
Static-2002R								
5-year								
I	Very low	1	0.074	7.4	0	0		
II	Below average	2	0.18	9.0	0	0		
III	Average	23	3.14	13.7	2	8.7	1.57	0.39, 6.28
IVa	Above average	53	11.3	21.3	5	9.4	2.26	0.94, 5.44
IVb	Well above average	105	35.1	33.4	22	21.0	**1.60**	**1.05, 2.42**
All		184	49.8	27.1	29	15.8	**1.72**	**1.19, 2.47**

*Note.* Bolded values are statistically significant at *p* < .05.

**Table 2.** Calibration (E/O index) of 15-year and 20-year sexual recidivism rates for Static-99R.

		*N*	Expected Recidivism		Observed Recidivism		E/O	95% CI
			*n* of Recidivists (E)	Proportion (E)	*n* of Recidivists (O, SE)	Proportion (O)		
15-year								
I	Very low	1	0.11	11.0	0	0		
II	Below average	6	0.80	13.3	0	0		
III	Average	39	8.60	22.1	9.09 (2.65)	23.3	0.95	0.53, 1.68
IVa	Above average	54	17.4	32.3	14.5 (3.32)	26.9	1.20	0.77, 1.89
IVb	Well above average	90	41.2	45.8	47.5 (4.77)	52.8	0.87	0.71, 1.06
All		190	68.1	35.9	71.2 (6.74)	37.5	0.96	0.80, 1.15
20-year								
I	Very low	1	0.12	12.0	0	0		
II	Below average	6	0.86	14.4	0	0		
III	Average	39	9.36	24.0	9.09 (2.65)	23.3	1.03	0.58, 1.82
IVa	Above average	54	19.2	35.6	16.7 (3.48)	31.0	1.15	0.76, 1.73
IVb	Well above average	90	45.3	50.3	48.6 (4.77)	54.0	0.93	0.77, 1.13
All		190	74.8	39.4	74.5 (6.82)	39.2	1.00	0.84, 1.20

*Note.* Median follow-up time of 20.5 years, with 89% followed for at least 15 years.

**Table 3.** Calibration (E/O index) of 15-year and 20-year sexual recidivism rates for Static-2002R.

		*N/n*	Expected Recidivism		Observed Recidivism		E/O	95% CI
			*n* of Recidivists (E)	Proportion (E)	*n* of Recidivists (O, SE)	Proportion (O)		
15-year								
I	Very low	1	0.13	12.8	0	0		
II	Below average	2	0.31	15.5	0	0		
III	Average	23	5.22	22.7	5.00 (1.98)	21.7	1.04	0.48, 2.27
IVa	Above average	54	18.4	34.0	11.2 (3.01)	20.8	1.64	0.97, 2.77
IVb	Well above average	110	53.8	48.9	54.9 (5.30)	49.9	0.98	0.81, 1.19
All		190	77.9	41.0	71.2 (6.74)	37.5	1.09	0.91, 1.32
20-year								
I	Very low	1	0.139	13.9	0	0		
II	Below average	2	0.336	16.8	0	0		
III	Average	23	5.71	24.8	5.00 (1.98)	21.7	1.14	0.53, 2.48
IVa	Above average	54	20.2	37.4	13.5 (3.26)	25.0	1.49	0.93, 2.40
IVb	Well above average	110	60.3	54.8	55.9 (5.30)	50.9	1.08	0.89, 1.30
All		190	86.7	45.6	74.5 (6.82)	39.2	1.16	0.97, 1.39

*Note.* Median follow-up time of 20.5 years, with 89% followed for at least 15 years.

When calibration was examined using logistic regression, none of the parameters were significantly different from the HRHN norms for either Static-99R (5-year, 10-year) or Static-2002R (5-year; see Table 4). Although there were no significant differences, there was a trend towards lower base rates (smaller *B0*’s) and better discrimination (higher *B1*’s) in the current HRHN sample compared to the HRHN norms. Contrary to expectation, the observed recidivism rates for our preselected subgroup were very close to the Routine/Complete norms (see Table 4). For five of the six comparisons with the Routine/Complete norms, the differences were less than expected by chance (*Q* < 1, *I*^*2*^ = 0), and in the other comparison the variability was small (*I*^*2*^ = 12.5%).

**Table 4.** Comparison of Logistic Regression Parameters in the current sample to the High Risk/High Needs (HRHN) and Routine/Complete Norms for Static-99R and Static-2002R.

	*B* (*SE*)	% or Odds Ratio	Comparison with Current		
			*Q*_*between*_ (*df* = 1)	*p*	*I* ^2^
Static-99R					
5-year					
Intercept (*B0*_*2*_)					
Current	−2.725678 (0.474215)	6.1%			
HRHN	−2.063626 (0.153444)	11.3%	1.764	.184	43.3
Routine/Complete	−3.035568 (0.066871)	4.6%	0.419	.518	0.00
Slope (*B1*)					
Current	0.300292 (0.108799)	1.35			
HRHN	0.250091 (0.042447)	1.28	0.185	.667	0.00
Routine/Complete	0.372803 (0.021437)	1.45	0.428	.513	0.00
10-year					
Intercept (*B0*_*2*_)					
Current	−2.155873 (0.386044)	10.4%			
HRHN	−1.442304 (0.186037)	19.1%	2.773	.096	63.9
Routine/Complete	−2.556535 (0.118530)	7.2%	0.984	.321	0.00
Slope (*B1*)					
Current	0.372137 (0.092412)	1.45			
HRHN	0.230673 (.056260)	1.26	1.710	.191	41.5
Routine/Complete	0.364911 (0.037367)	1.44	0.005	.942	0.00
Static-2002R					
5-year					
Intercept (*B0*_*3*_)					
Current	−2.865787 (0.538280)	5.4%			
HRHN	−1.877664 (0.173236)	13.3%	3.054	.081	67.2
Routine/Complete	−2.623861 (0.109268)	6.8%	0.194	.660	0.00
Slope (*B1*)					
Current	0.276160 (0.105218)	1.32			
HRHN	0.217648 (0.050133)	1.24	0.252	.616	0.00
Routine/Complete	0.395056 (0.036062)	1.48	1.143	.285	12.5

*Note.* The sample size for current analyses were as follows: Static-99R/5-year 29/184, Static-99R/10-year 53/182, and for Static-2002R/5-year 29/184 (number of recidivists/*N*). Static-99R norms from Lee and Hanson (2021); Static-2002R norms from Hanson et al. (2016).

**Figure 1.**
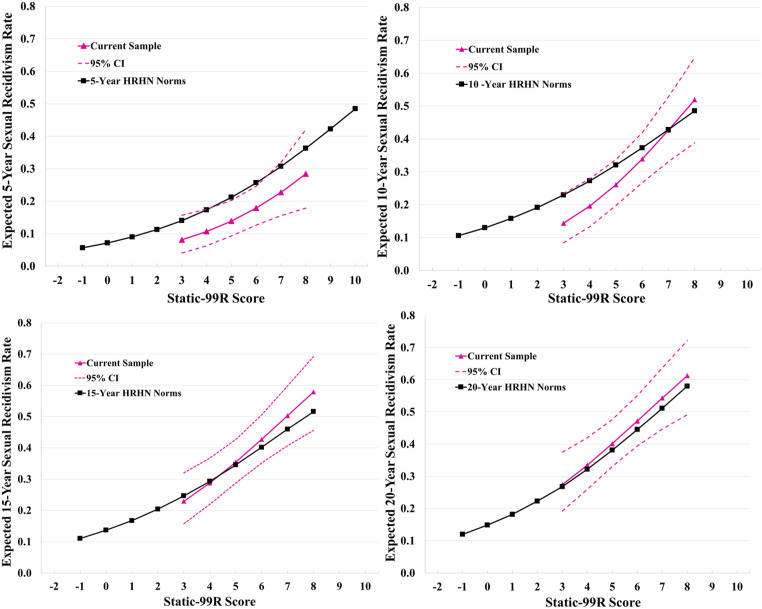
Logistic curves for 5, 10, 15, and 20-year sexual recidivism rates of the current sample with the Static-99R High Risk/High Need (HRHN) norms.

Figure 1 presents a visual representation of the calibration of Static-99R against the HRHN norms for 5 years, 10 years, 15 years, and 20 years. The Static-99R observed values are smoothed logistic regression curves (with 95% confidence intervals) plotted for each score that was populated by at least 10 cases. As can be seen from Figure 1, the Static-99R norms tended to overestimate the observed sexual recidivism rates at the shorter follow-up periods (5 years and 10 years); however, they were closely aligned with the observed values for the longer follow-up periods (15 years and 20 years). Calibration plots for Static-2002R for 5 years, 15 years, and 20 years are presented in Figure S1 (see online supplemental materials). The Static-2002R HRHN norms overestimated the observed recidivism rates at 5 years, whereas the observed rates were close to the expected rates at 15 years and 20 years. We did not calculate calibration for Static-2002R at 10-year follow-up because there are no 10-year HRHN norms asserted by the test developers (see Helmus et al., 2021b).

**Figure 2.**
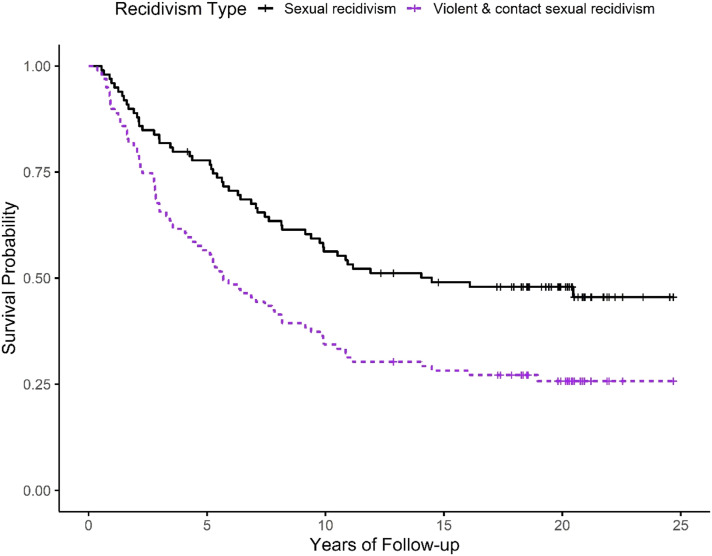
Sexual and violent (including contact sexual) recidivism rates for individuals identified as well above average risk to reoffend (Kaplan-Meier survival curves; *n* = 99).

The next analyses examined the observed sexual and violent recidivism rates for the 99 individuals in the *well above average* subgroup (i.e., individuals with SRA scores > 4 and Static-99R and Static-2002R scores in the *well above average* range: Level IVb). In this subgroup, 52.5% (52/99) were known to have sexually reoffended during the follow-up period. Based on Kaplan-Meier survival analysis (see Figure 2), the sexual recidivism rates were 22.2% at 5 years, 43.7% at 10 years, and 52.1% at 20 years. There was only one new sexual recidivism event after 17 years (at 20.5 years). For violent recidivism, 73.7% (73/99) reoffended with a violent or contact sexual offence during the full follow-up period (ragged follow-up). Based on Kaplan-Meier survival analysis the violent recidivism rates were 44.4% at 5 years, 67.7% at 10 years, and 74.3% at 20 years. There was only one new violent recidivism event after 17 years (at 19 years). The sexual recidivism rates for the highest risk individuals (top 1%; Static-99R > 7; Static-2002R > 9) were in the range of 60%–70%. Four received a Dangerous Offender designation for their recidivism event, and an additional 18 received Long Term Supervision Orders (a preventive detention provision with a determinant sentence).

The observed sexual recidivism rates per score on the Static-99R and Static-2002R risk tools are provided in the online Supplemental Materials (see Table S1, Table S2, Table S3, and Table S4). The 5-year and 10-year rates are based on fixed follow-up periods; the 15-year and 20-year rates are based on Kaplan-Meier survival analysis. The supplemental tables also provide a line of best fit for these observed values, smoothed using logistic regression. For the purpose of the logistic regression fit, the observed values from the Kaplan-Meier survival analyses were rounded to integers.

## Discussion

This study examined the extent to which it is possible to identify, in advance, individuals who present a high risk for sexual recidivism. Using a structured approach to risk assessment and long-term follow-up, we identified a group whose observed sexual recidivism rates were above 50%. Although there is no universal threshold for high risk, sexual recidivism rates in this range are clearly problematic and would presumptively meet the “more likely than not” threshold enshrined in certain civil commitment statutes in the USA. Furthermore, three out of four individuals in this *well above average* group reoffended with a nonsexual violent or contact sexual offence, indicating substantial risk of a serious personal injury offence (a criteria of Canada’s Dangerous Offender provision). Given that a certain proportion of the convictions for nonsexual violence will be for crimes with a sexual motivation (e.g., kidnapping), the actual rate of sexual recidivism will be higher than the rate estimated when only counting offences that are explicitly sexual.

The observed sexual recidivism rates would also underestimate the true rates because not all offences are detected. Although the size of the gap between detected and undetected reoffenders remains a topic of debate (Abbott, 2020; Lave et al., 2021; Scurich & John, 2019), there is no question that nonreporting obscures the true recidivism rate. We believe, however, that the gap between detected and undetected offenders should decrease as the follow-up time increase. Even if the likelihood of detection per offence is low, individuals who repeatedly offend are likely to eventually get caught.

When the decision threshold concerns lifetime risk, most recidivism rate estimates are limited because they are based on shorter follow-up periods (e.g., 5 years, 10 years). Consequently, evaluators must extrapolate from the reported rates to lifetime rates. Hanson, Thornton and colleagues (Hanson et al., 2018; Thornton et al., 2021) have argued that for sexual recidivism the 20-year rates closely approximate the lifetime rates because the hazards of sexual recidivism become vanishingly small for individuals who remain 20 years sexual offence free in the community. This assumption was supported in the current study. The residual hazard of sexual recidivism was small after 15 years sexual offence free. In the full sample of 377 individuals, the residual hazard was only 3.4% after 15 years, compared to 29.2% between time of release and 15 years. Among the 181 individuals who remained sexual offence free for more than 20 years, there were only two subsequent sexual recidivism events (at 20.5 and 21.4 years). Even though there were few individuals followed for more than 25 years, the consistency of the current findings with previous research (Hanson et al., 2018; Moore, 2018) suggests that the observed 20-year sexual recidivism rates in the current study reasonably approximate the rates that would have been observed if all the individuals had been followed to the end of their lives.

A related goal was to evaluate the predictive accuracy (calibration) of the Static-99R and Static-2002R sexual recidivism prediction tools for high risk/high need (HRHN) samples. The results were mixed. The HRHN norms substantially overestimate the observed sexual recidivism rates for the shorter follow-up periods (5 years) for both Static-99R and Static-2002R, but were close to the observed values at 15 years and 20 years. This pattern was opposite to our expectations. We had greater a priori confidence in the 5-year and 10-year rates given that they required fewer inferences than the 15-year and 20-year norms. Nevertheless, the good calibration of the STATIC risk tools at 20-year follow-up should be reassuring to evaluators wishing to use these measures to estimate long-term (e.g., lifetime) sexual recidivism rates.

Even though the current study broadly supported the high risk/high needs norms for the STATIC measures, the observed values also fit the norms for routine/complete samples. This pattern could be explained, in retrospect, by the unusually high STATIC scores in this sample. In routine/complete samples, the median Static-99R value is usually 2 (*SD* = 2.1; Hanson et al., 2012), whereas the median value in the current study was 5; furthermore, 76% of the sample (144/190) had a Static-99R score in the *above average* range (4 or higher). Conversely, there were almost no individuals in the *very low* (1/190) or *below average* (6/190) risk levels. The logistic regression equations that define the STATIC norms assume that Static-99R and Static-2002R show greater discrimination in routine/complete samples than in high risk/high need samples, which results in routine/complete samples having similar expected rates for high scores (Hanson et al., 2016). Consequently, the expected values are substantially similar for both sets of recidivism rate norms (routine/complete and high risk/high need) for the Static-99R values populated in this study.

Most studies find a consistent decline in sexual recidivism risk for each year that the individual remains offence-free in the community (Hanson et al., 2018). In previous meta-analyses, the sexual recidivism rates between 5 years and 10 years were approximately half the sexual recidivism rates observed between time of release and 5 years (Hanson et al., 2014a, 2018; Harris & Hanson, 2004). In contrast, the current study found similar sexual recidivism hazard rates during the first 10 years, followed by a sharp decline in risk for subsequent years (see Figure 2). This pattern may be a random feature of this dataset, or a consequence of delayed processing of sexual assault cases in the criminal justice system (i.e., conviction dates are often years after offence dates). It is possible, however, that it is related to protective effects of community supervision. All the individuals in the original study were on community supervision at that time, and many were nominated for the study by their supervising officers because of concerns about sexual recidivism risk. Although the risk reduction effects of community supervision remain an open research topic, the current study is not the first to show relatively flat hazard functions during the first 10 years for individuals who started then exited community supervision (Ducro et al., 2020; Zgoba et al., 2012).

When the sexual recidivism outcome was expanded to include sexually motivated community supervision violations, the recidivism rate during the first 5 years (24.4%, *n* = 190) was larger (as expected) than the rate during years 5–10 (13.9%). Given that the circumstances of the recidivism events were not always known, some of the community supervision violations could have been for sexual crimes. Consequently, in addition to suppressing actual recidivism, community supervision could be suppressing the apparent reconviction rate (i.e., some sexual crimes could be sanctioned by return to prison rather than new charges). Including violations in the definition of sexual recidivism would move the observed rates away from the routine/complete norms and closer to the HRHN norms. Nevertheless, we recommend against including community supervision violations in the definition of sexual recidivism because the actual behaviours were unknown.

### Limitations

Missing records is a common challenge for long term follow-up studies, and the current study was no exception. In 1997, official criminal history records were available for all 409 cases identified for the original study, whereas in 2017 recidivism information was only available for 377 cases (92.2%). The reasons for missing records are not known, and would vary over the course of the 20+ year follow-up time. Nevertheless, our previous experience with the RCMP CPIC records is that they are unlikely to go missing for individuals who remain in the criminal justice system (i.e., the recidivists). In the *well above average* subgroup there were 99 individuals with codable recidivism information, of which 52 were known to have reoffended with a sexual crime (raw recidivism rate of 52.5% = 52/99). As well, there were an additional 6 individuals without codable recidivism information (four without FPS records plus two with FPS records that lacked the necessary released dates). If all six individuals in this group were considered nonrecidivists, the observed estimate would decrease to 49.5% (52/105); if all six were recidivists, the estimate would increase to 55.2% (58/105). Given that both values are close to unadjusted value of 52.5%, it is unlikely that missing records introduced substantial bias.

Another limitation was that the SRA scheme used to identify a high risk/high need subsample was applied retrospectively using variables previously coded. Although the dataset was sufficiently rich that the SRA implementation was plausible, it was not ideal. In particular, as compared to typical implementations of the SRA Need Framework, the present implementation was based heavily on observations made by supervising agents, and so is more oriented to recent functioning than is desirable where the intention is to assess long-term vulnerabilities. Additionally, for the most part, the variables being integrated were not from established measures with established reliability and validity.

A related limitation is that the threshold for HRHN (SRA score of 4) was unique to this study. Although plausibly aligned with the STATIC definition, there were no established norms to justify this specific score for this particular operationalization of the SRA system. In order to explore the potential influence of the choice of threshold, it was varied from 4 (approximately the 50^th^ percentile), to 3 (30^th^ percentile), and to 5 (70^th^ percentile). The raw sexual recidivism rate of the *well above average* group remained unchanged: 4 (52.5% 52/99), 3 (52.5% 52/99) and 5 (52.5%, 32/61). Consequently, it is unlikely that minor changes to the SRA threshold would substantially influence the overall findings.

### Implications for Practice

The study provided general support for the High Risk/High Need (HRHN) recidivism rate norms of the Static-99R and Static-2002R sexual recidivism risk tools. Although these norms overestimated the observed recidivism rates at 5-years, they were well calibrated for the longer follow-up periods. The poor calibration at 5 years, on the other hand, suggests that evaluators should be cautious when making inferences about the likelihood of recidivism during this time period, particularly if the individual in question has external protective factors, such as ongoing treatment and a credible supervision plan.

The use of the HRHN norms requires an initial judgement that the individual has unusually high levels of criminogenic needs (Hanson et al., 2016). The specific method used in the current study, however, cannot be directly implemented in applied practice because it was based on archival research data. Instead, prudent evaluators should make the determination that the individual is a member of a presumptively high-risk group based on a validated measure of criminogenic needs, such as the STABLE-2007 (Brankley et al., 2021), and the VRS-SO (Olver et al., 2007), or the forensic version of the SRA (e.g., SRA-FV; Thornton & Knight, 2015),

### Implications for Research

More studies of HRHN samples are needed. Static-99R studies of routine/complete samples are sufficiently common to support regular updates of the recidivism rate norms for this reference group (Lee & Hanson, 2021). To our knowledge, this is the first HRHN validity study of either Static-99R or Static-2002R since 2008 (Hanson et al., 2016). The total number of HRHN samples remains small (*k* = 6 for Static-99R; *k* = 3 for Static-2002R, including the current study).

We hypothesized that community supervision suppressed recidivism during the first 5 years following release (i.e., flattened the hazard rates). This speculation, however, was made without knowledge of the actual interventions received. Future research could advance understanding of effective interventions by monitoring the type and intensity of treatment and supervision efforts, and by monitoring changes in psychological and community adjustment (e.g., Babchishin & Hanson, 2020).

Further research is also needed on the effect of illness and mortality on long term recidivism rates. There is anecdotal evidence that exceptionally good health may mitigate the protective effect of advanced age on sexual recidivism risk (Mattek & Hanson, 2018). Much knowledge could be gained by data linkage studies that combined criminal justice, health, and mortality records. In particular, it would inform case management decisions about individuals previously identified as high risk who are now in their advanced old age.

## Conclusion

Preventative detention measures, such as Sexually Violent Person’s laws in the US and Dangerous Offender provisions in Canada, rest on the assumption that there are certain individuals who present a high risk for sexual recidivism. For such provisions to work, evaluators and decision-makers must be able to identify these high-risk individuals in advance. The current findings support these assumptions. Although the ethical and legal implications of preventive detention need to be carefully considered, there are individuals who present a significant, enduring risk to public safety. Furthermore, evaluators using existing technologies can identify this problematic subgroup with reasonable accuracy.

## Supplemental Material

Supplemental Material - Long Term Recidivism Rates Among Individuals at High Risk to Sexually Reoffend Supplemental Material for Long Term Recidivism Rates Among Individuals at High Risk to Sexually Reoffend by R. Karl Hanson, Seung C. Lee, and David Thornton in Sexual Abuse

Supplemental Material - Long Term Recidivism Rates Among Individuals at High Risk to Sexually Reoffend Supplemental Material for Long Term Recidivism Rates Among Individuals at High Risk to Sexually Reoffend by R. Karl Hanson, Seung C. Lee, and David Thornton in Sexual Abuse

## Appendix

Assigning standardized risk levels based on averaging Static-99R and Static-2002R scores.

Previous research has indicated that the average of Static-99R and Static-2002R scores has greater predictive accuracy than either measure on its own (Babchishin et al., 2012a; Lehmann et al., 2013). It is not obvious, however, how evaluators should calculate the average for specific cases because the raw scores of these measures are in different metrics. Consistent with previous research, in the current study the scores were averaged in the metric of logged hazard ratios (Babchishin et al., 2012a, 2012b; Lehmann et al., 2013). Hazard ratios were privileged because relative risk inferences are stable features of these prediction tools (Helmus et al., 2012a). The hazard ratios were log transformed because the effect of a one-point increase in STATIC scores is linear for logged hazard ratios (not for raw hazard ratios).

In the STATIC norms, logged hazard ratios are presented from Cox regression survival analysis, centered on the median values (β = 0.3318; median of 2 for Static-99R; β = .322, median of 3 for Static-2002R; Helmus et al., 2021b). In order to translate the averages into standardized risk levels, the boundaries between the risk levels were calculated as the midpoint between the hazards for adjacent risk levels. Specifically, when identifying the boundary between Level IVa and IVb for Static-99R, the logged hazard ratio for a score of 5 (the top of Level IVa) was 0.9954 and the logged hazard ratio for a score of 6 (the bottom of Level IVb) was 1.3272; consequently, the boundary was 1.1613 = (0.9954 + 1.3272)/2. For Static-2002R, the boundary between Level IVa and Level IVb was similar (1.127) but not identical. Consequently, for the purpose of assigning risk levels, the boundary was calculated as the average of the boundaries for Static-99R and Static-2002R (i.e., logged hazard ratio of 1.14415; hazard ratio of *e*^1.14415^ = 3.140).

## References

[bibr1-10790632221139166] Abbott B. R. (2020). Illuminating the dark figure of sexual recidivism. Behavioral Sciences & the Law, 38(6), 543–558. 10.1002/bsl.2494 33230891

[bibr2-10790632221139166] Aelick C. A. Babchishin K. M. Harris A. J. R. (2020). Severe mental illness diagnoses and their association with reoffending in a sample of men adjudicated for sexual offences. Sexual Offending: Theory, Research, and Prevention*, *15(1), Article e3123. 10.5964/sotrap.3123

[bibr3-10790632221139166] Alper M. Durose M. R. (2019). Recidivism of sex offenders released from state prison: A 9-year follow-up (2005-14). Special report NCJ 251773. Bureau of Justice Statistics, Office of Justice Programs, U.S. Department of Justice.

[bibr4-10790632221139166] Babchishin K. M. Hanson R. K. (2020). Monitoring changes in risk of reoffending: A prospective study of 632 men on community supervision. Assessment, 19(4), 886–898. 10.1037/ccp0000601. 32804519

[bibr5-10790632221139166] Babchishin K. M. Hanson R. K. Helmus L. (2012a). Communicating risk for sex offenders: Risk ratios for Static-2002R. Sexual Offender Treatment, 12(2), 1–12.

[bibr6-10790632221139166] Babchishin K. M. Hanson R. K. Helmus L. (2012b). Even highly correlated measures can add incrementally to predicting recidivism among sex offenders. Assessment, 19(4), 442–461. 10.1177/1073191112458312. 22946101

[bibr7-10790632221139166] Baudin C. Nilsson T. Sturup J. Wallinius M. Andiné P. (2021). A Static-99R validation study on individuals with mental disorders: 5 to 20 years of fixed follow-up after sexual offenses. Frontiers in Psychology, 12. 10.3389/fpsyg.2021.625996. PMC7884330 33603706

[bibr8-10790632221139166] Blais J. Bonta J. (2015). Tracking and managing high risk offenders: A Canadian initiative. Law and Human Behavior, 39(3), 253–265. 10.1037/lhb0000109 25365473

[bibr9-10790632221139166] Boccaccini M. T. Rice A. K. Helmus L. M. Murrie D. C. Harris P. B. (2017). Field validity of Static-99/R scores in a statewide sample of 34,687 convicted sexual offenders. Psychological Assessment, 29(6), 611–623. 10.1037/pas0000377 28594205

[bibr10-10790632221139166] Borenstein M. Hedges L. V. Higgins J. P. T. Rothstein H. R. (2021). Introduction to meta-analysis (2nd ed.). Wiley.

[bibr11-10790632221139166] Bourgon G. Mugford R. Hanson R. K. Coligado M. (2018). Offender risk assessment practices vary across Canada. Canadian Journal of Criminology and Criminal Justice, 60(2), 167–205. 10.3138/cjccj.2016-0024

[bibr12-10790632221139166] Brankley A. E. Babchishin K. M. Hanson R. K. (2021). STABLE-2007 demonstrates predictive and incremental validity in assessing risk-relevant propensities for Sexual offending: A meta-analysis. Sexual Abuse, 33(1), 34–62. 10.1177/1079063219871572 31516097

[bibr13-10790632221139166] Cohen J. (1988). Statistical power analysis for the behavioral sciences. Routledge Academic.

[bibr14-10790632221139166] Criminal Code of Canada . (1985). RSC. c.C-46.

[bibr15-10790632221139166] Ducro C. Telle E. Pham T. (2020). Recidivism rates of sex offenders released from the French Belgian Judicial System. Acta Psychiatrica Belgica, 120(1), 43–49.

[bibr16-10790632221139166] Gonçalves L. C. Gerth J. Rossegger A. Noll T. Endrass J. (2020). Predictive validity of the Static-99 and Static-99R in Switzerland. Sexual Abuse, 32(2), 203–219. 10.1177/1079063218821117 30623752

[bibr17-10790632221139166] Hanson R. K. (2017). Assessing the calibration of actuarial risk scales: A primer on the E/O index. Criminal Justice and Behavior, 44(1), 26–39. 10.1177/0093854816683956

[bibr18-10790632221139166] Hanson R. K. (2022). Prediction statistics for psychological assessment*.* American Psychological Association.

[bibr19-10790632221139166] Hanson R. K. Babchishin K. M. Helmus L. M. Thornton D. Phenix A. (2017). Communicating the results of criterion referenced prediction measures: Risk categories for the Static-99R and Static-2002R sexual offender risk assessment tools. Psychological Assessment, 29(5)*,* 582–597. 10.1037/pas0000371 27618202

[bibr20-10790632221139166] Hanson R. K. Bussière M. T. (1998). Predicting relapse: A meta-analysis of sexual offender recidivism studies. Journal of Consulting and Clinical Psychology, 66(2), 348–362. 10.1037/0022-006X.66.2.348 9583338

[bibr21-10790632221139166] Hanson R. K. Harris A. J. R. (2000a). The Sex Offender Need Assessment Rating (SONAR): A method for measuring change in risk levels. User report 2000-01. Department of the Solicitor General of Canada.

[bibr22-10790632221139166] Hanson R. K. Harris A. J. R. (2000b). Where should we intervene? Dynamic predictors of sex offense recidivism. Criminal Justice and Behavior, 27(1), 6–35. 10.1177/0093854800027001002.

[bibr23-10790632221139166] Hanson R. K. Harris A. J. R. Helmus L. Thornton D. (2014a). High risk sex offenders may not be high risk forever. Journal of Interpersonal Violence, 29(15), 2792–2813. 10.1177/0886260514526062. 24664250

[bibr24-10790632221139166] Hanson R. K. Harris A. J. R. Letourneau E. Helmus L. M. Thornton D. (2018). Reductions in risk based on time offense free in the community: Once a sexual offender, not always a sexual offender. Psychology, Public Policy and Law, 24(1), 48–63. 10.1037/law0000135

[bibr25-10790632221139166] Hanson R. K. Helmus L. Harris A. J. R. (2015). Assessing the risk and needs of supervised sexual offenders: A prospective study using STABLE-2007, Static-99R and Static-2002R. Criminal Justice and Behavior, 42(12), 1205–1224. 10.1177/0093854815602094

[bibr26-10790632221139166] Hanson R. K. Helmus L. Thornton D. (2010). Predicting recidivism among sexual offenders: A multi-site study of Static-2002. Law and Human Behavior, 34(3), 198–211. 10.1007/s10979-009-9180-1 19536646

[bibr27-10790632221139166] Hanson R. K. Lloyd C. D. Helmus L. Thornton D. (2012). Developing non-arbitrary metrics for risk communication: Percentile ranks for the Static-99/R and Static-2002/R sexual offender risk scales. International Journal of Forensic Mental Health, 11(1), 9–23. 10.1080/14999013.2012.667511

[bibr28-10790632221139166] Hanson R.K. Lunetta A. Phenix A. Neely J. Epperson D. (2014b). The field validity of Static-99R sex offender risk assessment tool in California. Journal of Threat Assessment and Management, 1(2), 102–117. 10.1037/tam0000014.

[bibr29-10790632221139166] Hanson R. K. Morton-Bourgon K. E. (2009). The accuracy of recidivism risk assessments for sexual offenders: A meta-analysis of 118 prediction studies. Psychological Assessment, 21(1), 1–21. 10.1037/a0014421 19290762

[bibr30-10790632221139166] Hanson R. K. Thornton D. (2000). Improving risk assessments for sex offenders: A comparison of three actuarial scales. Law and Human Behavior, 24(1), 119–136. 10.1023/A:1005482921333 10693322

[bibr31-10790632221139166] Hanson R. K. Thornton D. (2003). Notes on the development of static-2002. (Corrections research user report No. 2003-01). Department of the Solicitor General of Canada. https://www.publicsafety.gc.ca/cnt/rsrcs/pblctns/nts-dvlpmnt-sttc/nts-dvlpmnt-sttc-eng.pdf

[bibr32-10790632221139166] Hanson R. K. Thornton D. Helmus L.-M. Babchishin K. M. (2016). What sexual recidivism rates should be associated with Static-99R and Static-2002R scores? Sexual Abuse: A Journal of Research and Treatment, 28(3), 218–252. 10.1177/107906321557410 25810478

[bibr33-10790632221139166] Harris A. J. R. Hanson R. K. (2004). Sex offender recidivism: A simple question *.* Corrections Users Report No. 2004-03. Public Safety and Emergency Preparedness Canada.

[bibr34-10790632221139166] Helmus L. Hanson K. (2007). Predictive validity of the Static-99 and Static-2002 for sex offenders on community supervision. Sexual Offender Treatment, 2, 1–14.

[bibr40-10790632221139166] Helmus L. M. Lee S. C. Phenix A. Hanson R. K. Thornton D. (2021b). *Static-99R & Static-2002R evaluators’ workbook.* SAARNA: The Society for the Advancement of Actuarial Risk Need Assessment. www.saarna.org

[bibr37-10790632221139166] Helmus L. M. Babchishin K. M. (2017). Primer on risk assessment and the statistics used to evaluate its accuracy. Criminal Justice and Behavior, 44(1), 8–25. 10.1177/0093854816678898

[bibr38-10790632221139166] Helmus L. M. Hanson R. K. Murrie D. C. Zabarauckas C. L. (2021a). Field validity of Static-99R and STABLE-2007 with 4,433 men serving sentences for sexual offences in British Columbia: New findings and meta-analysis. Psychological Assessment, 33(7), 581–595. 10.1037/pas0001010. 34014750

[bibr35-10790632221139166] Helmus L. Hanson R. K. Thornton D. Babchishin K. M. Harris A. J. R. (2012a). Absolute recidivism rates predicted by Static-99R and Static-2002R sex offender risk assessment tools vary across samples: A meta-analysis. Criminal Justice and Behavior, 39(9), 1148–1171. 10.1177/0093854812443648.

[bibr39-10790632221139166] Helmus L. M. Kelley S. M. Frazier A. Fernandez Y. M. Lee S. C. Rettenberger M. Boccaccini M. T. (2022). Static-99R: Strengths, limitations, predictive accuracy meta-analysis, and legal admissibility review. Psychology, Public Policy, and Law, 28(3), 307–331. 10.1037/law0000351.

[bibr36-10790632221139166] Helmus L. Thornton D. Hanson R. K. Babchishin K. M. (2012b). Improving the predictive accuracy of Static-99 and Static-2002 with older sex offenders: Revised age weights. *Sexual Abuse*. A Journal of Research and Treatment, 24(1), 64–101. 10.1177/1079063211409951. 21844404

[bibr41-10790632221139166] Jung S. Ennis L. Hermann C. A. Pham A. T. Choy A. L. Corabian G. Hook T. (2017). An evaluation of the reliability, construct validity, and factor structure of the Static-2002R. International Journal of Offender Therapy and Comparative Criminology, 61(4), 464–487. 10.1177/0306624X15595228 26169567

[bibr42-10790632221139166] Kahneman D. Sibony O. Sunstein C. R. (2021). Noise: A flaw in human judgment. Little, Brown Spark.

[bibr43-10790632221139166] Kelley S. M. Ambroziak G. Thornton D. Barahal R. M. (2020). How do professionals assess sexual recidivism risk? An updated survey of practices. Sexual Abuse, 32(1), 3–29. 10.1177/1079063218800474 30244649

[bibr44-10790632221139166] Knighton J. C. Murrie D. C. Boccaccini M. T. Turner D. B. (2014). How likely is “likely to reoffend” in sex offender civil commitment trials? Law and Human Behavior, 38(3), 293–304. 10.1037/lhb0000079 24885113

[bibr45-10790632221139166] Laporte L. Poulin B. Marleau J. Roy R. Webanck T. (2003). Filicidal women: Jail or psychiatric ward? The Canadian Journal of Psychiatry, 48(2), 94–98. 10.1177/070674370304800205 12655906

[bibr46-10790632221139166] Lave T. R. Prescott J. J. Bridges G. (2021). The problem with assumptions: Revisiting “The dark figure of sexual recidivism”. Behavioral Sciences & the Law, 39(3), 279–306. 10.1002/bsl.2508 34125965

[bibr47-10790632221139166] Lee S. C. Hanson R. K. (2021). Updated 5-year and new 10-year sexual recidivism rate norms for Static-99R with routine/complete samples. Law and Human Behavior, 45(1), 24–38. 10.1037/lhb0000436 33734747

[bibr48-10790632221139166] Lee S. C. Hanson R. K. Blais J. (2020). Predictive accuracy of the Static-99R and Static-2002R risk tools for identifying Indigenous and White individuals at high risk for sexual recidivism in Canada. Canadian Psychology/Psychologie Canadienne, 61(1), 42–57. 10.1037/cap0000182

[bibr49-10790632221139166] Lee S. C. Hanson R. K. Fullmer N. Neeley J. Ramos K. (2018). The predictive validity of static-99R over 10 years for sexual offenders in California: 2018 update. State Authorized Risk Assessment Tools for Sex Offenders (SARATSO). http://saratso.org/pdf/Lee_Hanson_Fullmer_Neeley_Ramos_2018_The_Predictive_Validity_of_S_.pdf

[bibr50-10790632221139166] Lee S. C. Restrepo A. Satariano A. Hanson R. K. (2016). The predictive validity of static-99R for sex offenders in California: 2016 update. State Authorized Risk Assessment Tools for Sex Offenders (SARATSO). http://saratso.org/pdf/ThePredictiveValidity_of_Static_99R_forSexualOffenders_inCalifornia_2016v1.pdf

[bibr51-10790632221139166] Lehmann R. J. B. Hanson R. K. Babchishin K. Gallasch-Nemitz F. Biedermann J. Dahle K.-P. (2013). Interpreting multiple risk scales for sex offenders: Evidence for averaging. Psychological Assessment, 25(3)*,* 1019–1024. 10.1037/a0033098 23730829

[bibr52-10790632221139166] Mann R. E. Hanson R. K. Thornton D. (2010). Assessing risk for sexual recidivism: Some proposals on the nature of psychologically meaningful risk factors. Sexual Abuse: A Journal of Research and Treatment, 22(2), 191–217. 10.1177/1079063210366039 20363981

[bibr53-10790632221139166] Mattek R. Hanson R. K. (2018). Committed as a violent sexual predator in his 10th decade: A case study. Archives of Sexual Behavior, 47(2), 543–550*.* 10.1007/s10508-017-1041-2 28828586

[bibr54-10790632221139166] McGrath R. J. Lasher M. P. Cumming G. F. (2012). The Sex Offender Treatment Intervention and Progress Scale (SOTIPS): Psychometric properties and incremental predictive validity with Static-99R. Sexual Abuse, 24(5), 431–458. 10.1177/1079063211432475. 22368161

[bibr55-10790632221139166] Moore L. (2018). Static risk assessment of sexual offenders in New Zealand: Predictive accuracy, classification of risk, and the moderating effect of time offence-free in the community. Doctoral Dissertation, Psychology, University of Canterbury.

[bibr56-10790632221139166] Neal T. M. Grisso T. (2014). Assessment practices and expert judgment methods in forensic psychology and psychiatry: An international snapshot. Criminal Justice and Behavior, 41(12), 1406–1421. https://doi.org/10.1177%2F0093854814548449

[bibr57-10790632221139166] Olver M. E. Kelley S. M. Johnson L. Wong S. C. P. (2020). Violence Risk Scale-Sexual Offense version (VRS-SO): Users’ workbook. https://psynergy.ca/vrs-so

[bibr58-10790632221139166] Olver M. E. Kelley S. M. Kingston D. A. Beggs Christofferson S. M. Thornton D. Wong S. C. P. (2021). Incremental contributions of static and dynamic sexual violence risk assessment integrating Static-99R and VRS-SO common language risk levels. Criminal Justice and Behavior, 48(8), 1091–1110. 10.1177/0093854820974400

[bibr59-10790632221139166] Olver M. E. Wong S. C. P. Nicholaichuk T. Gordon A. (2007). The validity and reliability of the Violence Risk Scale-Sexual Offender version: Assessing sex offender risk and evaluating therapeutic change. Psychological Assessment, 19(3), 318–329. 10.1037/1040-3590.19.3.318 17845123

[bibr60-10790632221139166] Prentky R. A. Barbaree H. E. Janus E. S. (2015). Sexual predators: Society, risk, and the law. Routledge.

[bibr61-10790632221139166] Public Safety Canada . (2020). 2019 corrections and conditional release statistical overview. Public Safety Canada. https://www.publicsafety.gc.ca/cnt/rsrcs/pblctns/ccrso-2019/index-en.aspx#e3

[bibr62-10790632221139166] Raymond B. C. McEwan T. E. Davis M. R. Reeves S. G. Ogloff J. (2020). Investigating the predictive validity of Static-99/99R scores in a sample of older sexual offenders. Psychiatry, Psychology and Law, 28(1), 120–134. 10.1080/13218719.2020.1767714 PMC8451631 34552383

[bibr63-10790632221139166] R Core Team . (2020). R: A language and environment for statistical computing*.* R Foundation for Statistical Computing. https://www.R-project.org/

[bibr64-10790632221139166] Reeves S. G. Ogloff J. R. P. Simmons M. (2018). The predictive validity of the Static-99, Static-99R, and Static-2002/R: Which one to use? Sexual Abuse, 30(8), 887–907. 10.1177/1079063217712216 28597720

[bibr65-10790632221139166] Rice M. E. Harris G. T. (2005). Comparing effect sizes in follow-up studies: ROC Area, cohen's d, and r. Law and Human Behavior, 29(5), 615–620. 10.1007/s10979-005-6832-7 16254746

[bibr66-10790632221139166] Rockhill B. Byrne C. Rosner B. Louie M. M. Colditz G. (2003). Breast cancer risk prediction with a log-incidence model: Evaluation of accuracy. Journal of Clinical Epidemiology, 56(9), 856–861. 10.1016/S0895-4356(03)00124-0 14505770

[bibr67-10790632221139166] R. v Currie . (1997). 2. S.C.R. 260.

[bibr68-10790632221139166] Scurich N. John R. S. (2019). The dark figure of sexual recidivism. Behavioral Sciences & the Law, 37(2), 158–175. 10.1002/bsl.2400 30900348

[bibr69-10790632221139166] Singer J. Willet J. (2003). Applied longitudinal data analysis: Modeling change and event occurrence. Oxford University Press.

[bibr70-10790632221139166] Smid W. J. Kamphuis J. H. Wever E. C. Van Beek D. J. (2014). A comparison of the predictive properties of nine sex offender risk assessment instruments. Psychological Assessment, 26(3), 691–703. 10.1037/a0036616 24773035

[bibr71-10790632221139166] Stephens S. Cantor J. M. Goodwill A. M. Seto M. C. (2017). Multiple indicators of sexual interest in prepubescent or pubescent children as predictors of sexual recidivism. Journal of Consulting and Clinical Psychology, 85(6), 585–595. 10.1037/ccp0000194 28287801

[bibr72-10790632221139166] Tanguay D. (2004). Récits motards: Examen d'un conflit en milieu criminel. [Biker stories: Study of a conflict in the criminal underworld]. Unpublished Master’s thesis. École de Criminologie, Université de Montréal.

[bibr73-10790632221139166] Thornton D. (2002). Constructing and testing a framework for dynamic risk assessment. Sexual Abuse: A Journal of Research and Treatment, 14(2), 137–151. 10.1177/107906320201400205 11961888

[bibr74-10790632221139166] Thornton D. (2016). Structured risk assessment. In Phenix A. Hoberman H.M. (Eds.), Sexual offending: Predisposing antecedents, assessments and management (pp. 503–520). Springer.

[bibr75-10790632221139166] Thornton D. Hanson R. K. Kelley S. M. Mundt J. C. (2021). Estimating lifetime and residual risk for individuals who remain sexual offense free in the community: Practical applications. Sexual Abuse, 33(1), 3–33. 10.1177/1079063219871573 31478439

[bibr76-10790632221139166] Thornton D. Knight R. A. (2015). Construction and validation of SRA-FV need assessment. Sex Abuse: A Journal of Research and Treatment, 27(4), 360–375. 10.1177/1079063213511120 24379164

[bibr77-10790632221139166] Viallon V. Ragusa S. Clavel-Chapelon F. Bénichou J. (2009). How to evaluate the calibration of a disease risk prediction tool. Statistics in Medicine, 28(6), 901–916. 10.1002/sim.3517 19156698

[bibr78-10790632221139166] Viechtbauer W. (2010). Conducting meta-analyses in R with the *metafor* package. Journal of Statistical Software, 36(3), 1–48. 10.18637/jss.v036.i03

[bibr79-10790632221139166] Zgoba K. Miner M. Knight R. Letourneau E. Levenson J. Thornton D. (2012). A multi-state recidivism study using Static-99R and Static-2002R risk scores and tier guidelines from the Adam Walsh Act. U.S. Department of Justice Document No. 240099. https://www.ojp.gov/ncjrs/virtual-library/abstracts/multi-state-recidivism-study-using-static-99r-and-static-2002-risk

