# Supplementary material for: Long Term Recidivism Rates Among Individuals at High Risk to Sexually Reoffend
Source: Sex Abuse. 2022 Nov 16;36(1):3–32. doi: 10.1177/10790632221139166 (PMC11421192; doi:10.1177/10790632221139166)

Online Supplemental Materials for Hanson, Lee, & Thornton (2022)

Long term recidivism rates among individuals at high risk to sexually reoffend

Table S1

Sexual recidivism rates associated with Static-99R scores at 5 years and 10 years based on fixed follow-up

	5-year				10-year			
	Observed		Smoothed Logistic Regression		Observed		Smoothed Logistic Regression	
	%	*n* / *N*	%	95%CI	%	*n* / *N*	%	95%CI
**Scores**								
-3	0.0	0/1	1.44	[0.21, 9.26]	0.0	0/1	1.77	[0.36, 8.33]
-2	-	0/0	1.93	[0.35, 10.1]	-	0/0	2.55	[0.61, 9.95]
-1	0.0	0/1	2.59	[0.57, 10.9]	0.0	0/1	3.65	[1.06, 11.9]
0	0.0	0/5	3.47	[0.94, 11.9]	0.0	0/5	5.21	[1.81, 14.1]
1	11.1	1/9	4.63	[1.55, 13.0]	11.1	1/9	7.39	[3.08, 16.7]
2	0.0	0/7	6.15	[2.52, 14.2]	0.0	0/7	10.4	[5.15, 19.8]
3	8.7	2/23	8.13	[4.04, 15.7]	17.4	4/23	14.4	[8.44, 23.4]
4	18.2	4/22	10.7	[6.29, 17.5]	19.0	4/21	19.6	[13.3, 27.9]
5	10.3	3/29	13.9	[9.31, 20.2]	21.4	6/28	26.1	[19.7, 33.7]
6	13.2	5/38	17.9	[12.7, 24.6]	39.5	15/38	33.9	[26.7, 41.9]
7	19.2	5/26	22.7	[15.6, 31.9]	42.3	11/26	42.7	[33.1, 52.8]
8	41.2	7/17	28.4	[17.9, 42.0]	52.9	9/17	51.9	[38.8, 64.8]
9	40.0	2/5	34.9	[19.9, 53.7]	60.0	3/5	61.0	[44.2, 75.6]
10	0.0	0/1	42.0	[21.7, 65.4]	0.0	0/1	69.4	[49.5, 84.1]
Total	15.8	29/184			29.1	53/182		

Logit (5-year) = -2.725678[0.474215] + 0.300292[0.108799](Static-99R – 2); correlation of estimates = -.899396
Logit (10-year) = -2.155873[0.386044] + 0.372137[0.092412](Static-99R – 2); correlation of estimates = -0.894705
Note: In the above equations, the parameters are followed by their standard errors in brackets.

Table S2

Sexual recidivism rates associated with Static-99R score at 15 years and 20 years based on Kaplan-Meier survival analysis

	15-year					20-year			
	N	Observed		Smoothed Logistic Regression		Observed		Smoothed Logistic Regression	
		%	*n (n)*	%	95%CI	%	*n / N*	%	95%CI
**Scores**									
-3	1	0.0	0 (0)	3.12	[0.78, 11.6]	0.0	0(0)	3.44	[0.89, 1.24]
-2	0	-	0 (0)	4.38	[1.29, 13.8]	-	0(0)	4.82	[1.46, 14.7]
-1	1	0.0	0 (0)	6.11	[2.13, 16.3]	0.0	0(0)	6.71	[2.40, 17.4]
0	5	0.0	0 (0)	8.46	[3.48, 19.2]	0.0	0(0)	9.27	[3.90, 20.5]
1	9	11.1	1.0 (1)	11.6	[5.61, 22.5]	11.1	1.0 (1)	12.7	[6.27, 24.0]
2	7	14.3	1.0 (1)	15.7	[8.90, 26.3]	14.3	1.0 (1)	17.1	[9.88, 28.0]
3	23	30.7	7.1 (7)	21.0	[13.7, 30.7]	30.7	7.1 (7)	22.7	[15.1, 32.6]
4	23	22.4	5.2 (5)	27.4	[20.2, 35.9]	22.4	5.2 (5)	29.4	[22.1, 38.0]
5	31	30.0	9.3 (9)	34.9	[27.9, 42.5]	37.2	11.5 (12)	37.2	[30.1, 44.9]
6	38	50.1	19.0 (19)	43.2	[35.6, 51.2]	50.1	19.0 (19)	45.7	[37.9, 53.7]
7	26	50.0	13.0 (13)	52.0	[42.2, 61.5]	54.5	14.2 (14)	54.5	[44.7, 63.9]
8	18	55.6	10.0 (10)	60.6	[48.1, 71.8]	55.6	10.0 (10)	63.0	[50.6, 73.8]
9	7	81.0	5.7 (6)	68.6	[53.5, 80.6]	81.0	5.7 (6)	70.7	[56.0, 82.1]
10	1	0.0	0 (0)	75.6	[58.6, 87.2]	0.0	0 (0)	77.4	[61.1, 88.2]
Total	190	37.5				39.2			

Note: In the column *n*(*n*), *n* indicates the observed number of recidivists estimated by Kaplan-Meier survival analysis and (*n*) is *n* rounded to an integer (for logistic regression).
Logit (15 year) = -1.678214 [0.330643] + 0.351399 [0.082134] (Static-99R – 2). Correlation of estimates = -0.87679797
Logit (20 year) = -1.577884 [0.322587] + 0.351363 [0.081044] (Static-99R – 2). Correlation of estimates = -0.87252089
Note: In the above equations, the parameters are followed by their standard errors in brackets.

Table S3

Sexual recidivism rates associated with Static-2002R scores at 5 years and 10 years based on fixed follow-up

	5-year				10-year			
	Observed		Logistic Regression		Observed		Logistic Regression	
	%	*n / N*	%	95%CI	%	*n / N*	%	95%CI
**Scores**								
-1	0.0	0/1	1.85	[0.30, 10.7]	0.0	0/1	1.62	[0.33, 7.46]
0	-	0/0	2.43	[0.48, 11.4]	-	0/0	2.37	[0.59, 9.08]
1	0.0	0/2	3.17	[0.77, 12.2]	0.0	0/2	3.46	[1.03, 11.0]
2	0.0	0/7	4.14	[1.22, 13.1]	0.0	0/7	5.03	[1.79, 13.3]
3	14.3	1/7	5.39	[1.94, 14.1]	14.3	1/7	7.25	[3.09, 16.1]
4	11.1	1/9	6.98	[3.05, 15.2]	22.2	2/9	10.4	[5.25, 19.4]
5	5.0	1/20	9.00	[4.72, 16.5]	10.0	2/20	14.6	[8.70, 23.4]
6	12.1	4/33	11.5	[7.07, 18.3]	18.2	6/33	20.1	[13.9, 28.3]
7	14.8	4/27	14.7	[10.0, 20.9]	28.0	7/25	27.1	[20.6, 34.7]
8	22.2	6/27	18.5	[13.1, 25.4]	44.4	12/27	35.4	[27.9, 43.7]
9	9.7	3/31	23.0	[15.6, 32.6]	29.0	9/31	44.8	[34.7, 55.3]
10	60.0	9/15	28.2	[17.5, 42.3]	73.3	11/15	54.5	[40.9, 67.4]
11	0.0	0/5	34.2	[19.1, 53.3]	60.0	3/5	63.8	[46.8, 78.0]
Total	15.8	29/184			29.1	53/182		

Logit (5-year) = -2.865787 [0.538280] + 0.276160 [0.105218] (Static-2002R – 3). Correlation of estimates = -0.923317.
Logit (10-year) = -2.548537 [0.458494] + 0.389657 [0.091938] (Static-2002R – 3). Correlation of estimates = -0.925513.
Note: In the above equations, the parameters are followed by their standard errors in brackets.

Table S4

Sexual recidivism rates associated with Static-2002R scores at 15 years and 20 years based on Kaplan-Meier survival analysis

	N	15-year				20-year			
		Observed		Smoothed Logistic Regression		Observed		Smoothed Logistic Regression	
		%	*n (n)*	%	95%CI	%	*n / N*	%	95%CI
**Scores**									
-1	1	0.0	0 (0)	2.48	[0.61, 9.54]	0.0	0 (0)	3.18	[0.83, 11.5]
0	0	-	0 (0)	3.61	[1.05, 11.7]	-	0 (0)	4.52	[1.39, 13.8]
1	2	0.0	0 (0)	5.23	[1.80, 14.2]	0.0	0 (0)	6.39	[2.31, 16.5]
2	7	0.0	0 (0)	7.51	[3.06, 17.3]	0.0	0 (0)	8.95	[3.81, 19.6]
3	7	28.6	2.0 (2)	10.7	[5.13, 20.9]	28.6	2.0 (2)	12.4	[6.21, 23.3]
4	9	33.3	3.0 (3)	15.0	[8.45, 25.1]	33.3	3.0 (3)	16.9	[9.90, 27/5]
5	20	15.0	3.0 (3)	20.6	[13.5, 30.0]	15.0	3.0 (3)	22.7	[15.3, 32.3]
6	34	24.2	8.2 (8)	27.6	[20.5, 36.0]	30.7	10.4 (10)	29.7	[22.5, 38.2]
7	28	36.9	10.3 (10)	35.9	[28.9, 43.6]	40.8	11.4 (11)	37.9	[30.8, 45.5]
8	28	50.0	14.0 (14)	45.2	[37.2, 53.3]	50.0	14.0 (14)	46.8	[38.7, 55.0]
9	33	42.4	14.0 (14)	54.8	[44.6, 64.6]	42.4	14.0 (14)	55.9	[45.7, 65.6]
10	15	80.0	12.0 (12)	64.1	[51.2, 75.2]	80.0	12.0 (12)	64.6	[51.8, 75.5]
11	6	80.0	4.8 (5)	72.4	[57.3, 83.7]	80.0	4.8 (5)	72.4	[57.5, 83.6]
Total	190	37.5				39.2			

Logit (15-year) = -2.124851 [0.403935] + 0.386419 [0.084062] (Static-2002R – 3); Correlation of estimates = -0.91735935.
Logit (20-year) = -1.954604 [0.388157] + 0.365002 [0.081592] (Static-2002R – 3); Correlation of estimates = -0.91256369.
Note: In the above equations, the parameters are followed by their standard errors in brackets.

Supplemental Appendix

Constructing a Need Score based on the SRA Framework

The SRA Need framework proposes that psychological risk factors related to sexual recidivism can be understood as long-term vulnerabilities (LTVs) falling into four domains comprising Sexual Interests, Distorted Attitudes, Relational Style, and Self-Management (Thornton, 2016). To be regarded as long-term vulnerability, a problem needs to have been present in an enduring and pervasive way such that it is not manifested solely in one context or solely over a limited period of time. An SRA Need assessment should provide a balanced assessment of a range of long-term vulnerabilities from at least three of the four domains. Indicators for these needs should demonstrate appropriate validity and reliability.

The table below summarizes the long-term vulnerabilities falling within each domain.

**Domains**	**Sub-Domains**	**Long-term vulnerabilities (LTV)**
Sexual Interests	Sexual Preoccupation	Intense, Impersonal Sexual Interests<br>Sexual Coping<br>Diverse Outlets
	Offense-Related Sexual Interests	Sexual Preference for Children<br>Sexualized Violence
Distorted Attitudes	Victim Schema	Women as Deceitful/Malicious<br>Adversarial Sexual Attitudes<br>Child Abuse Supportive Beliefs
	Rights & Means Schema	Excessive Entitlement<br>Machiavellianism
Relational Style	Inadequate Relational Style	Dysfunctional Self-Evaluation<br>Emotional Congruence with Children
	Lack of Emotionally-Intimate Relationships with Adults	Lack of Sustained Marital-Type Relationships<br>Marital Relationships marred by repeated Violence/Infidelity
	Aggressive Relational Style	Callousness<br>Grievance Thinking<br>Poorly Managed Anger
Self-Management	Social Deviance	Lifestyle Impulsiveness<br>Resistance to Rules and Supervision
	Dysfunctional Coping	Poor Problem-Solving<br>Poor Emotional-Control

To construct an implementation for the current study the data dictionary was reviewed and variables classified according to their potential relevance to the constructs in the above table. Where alternative

descriptions were available variables which implied some persistence of the construct were chosen. Within each domain selected variables were subjected to exploratory factor analysis to group them into more homogeneous categories, better corresponding to the long-term vulnerabilities. For each domain at least two summary scores and levels of these were recoded so that they fell between 0 and 2. The summary scores for each domain were averaged to create a summary score for each domain and then these domain summary scores were summed to give a Need score running between 0 and 8.

The following table shows the within domain summary scores that resulted from this process.

**Domains**	**Within Domain Summary Scores**
Sexual Interests	Sexual Preoccupation<br>Sexual Interest in Children
Distorted Attitudes	Attitude to Children<br>Attitude to Women
Relational Style	Lack of Emotionally Intimate Relationships with Adults<br>Isolation<br>Angry Aggression
Self-Management	PCL-SV Factor Two (Social Deviance)<br>Resistance to Supervision

It was not possible to find an operationalization of Sexualized Violence as a long-term vulnerability with the available variables, nor were there appropriate indicators for Dysfunctional Coping, but otherwise coverage of the domains was fairly good.

This implementation has two substantive limitations. First, with the exception of the PCL-SV, none of the indicators are established measures with known reliability and validity. Secondly, with some exceptions, most ratings are heavily based on the conduct of individuals as seen through the eyes of supervising agents who saw them for a limited period of time. This latter limitation means they likely overemphasize how long-term vulnerabilities manifested themselves during recent periods of supervision.

Determining a Threshold Level of Need to Identify HRHN Individuals

Three considerations informed the selection of this threshold.

Conceptually we understand “high need” individuals as having multiple intense criminogenic needs. This can be contrasted with individuals whose criminogenic needs are less intense, or more circumscribed, or those whose offending resulted from transitory, situational, factors, and who therefore do not have sustained criminogenic needs. In SRA terms this concept suggests that “high need” individuals should have serious criminogenic needs from more than one domain and that if only two domains are involved then there should be serious problems in more than one criminogenic need within each domain.

For clinical practice, Thornton & Helmus (2021) have recommended using the HRHN norms when an individual’s score on a dynamic instrument is equal to or exceeds the mean score typically found in HRHN samples. Hanson and Thornton (2012) reported a review of mean scores in HRHN samples of

27.24, 3.26. and 14.70 for the VRS-SO Initial Dynamic Risk scale, the SRA-FV, and the STABLE-2007. Although these means vary substantially, resulting from how ratings are scaled in the different instruments, in each case the HRHN sample mean is close to half the maximum possible score on the instrument. The maximum on the VRS-SO Initial Dynamic Risk scale is 51, for SRA-FV it is 6, and for STABLE-2007 it is 26. Thus, in each case the means in HRHN samples are close to but just a little above half the maximum possible score on the instrument. This suggests that the threshold adopted on our instrument should correspond approximately to half the maximum possible score.

The threshold adopted should be consistent with selecting a higher need group while also assigning sufficient numbers to that group to permit statistical analyses. This suggests that the threshold adopted should be close to the sample median.

These considerations do not fully constrain what the threshold should be but they do provide guidance. The actual threshold adopted was a score of at least 4. Such a score can be obtained by having serious, multi-need, problems within two domains; having a serious single-need problem in all four domains; or some mixture of serious, single and multi-need, problems in three domains. It therefore is consistent with the first consideration. It corresponds to half the maximum possible score, so it is consistent with the second consideration. And it is close to the median found in the complete sample which was 4.2. It is therefore consistent with the third principle.

Importantly, both construction of the present SRA Need score and selection of the threshold were done while blind to the recidivism data.

Figure S1

Logistic curves for 5, 15, and 20-year sexual recidivism rates of a current sample with the Static-2002R High Risk/High Need norms

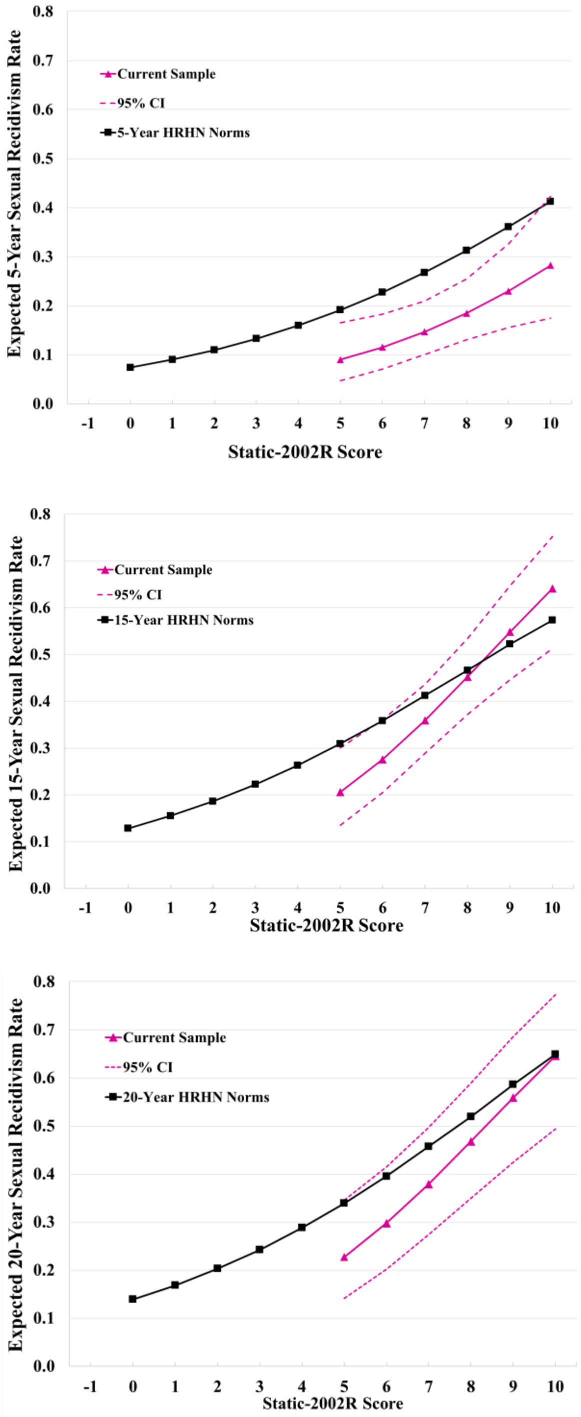

Supplement: Supplemental Material - Long Term Recidivism Rates Among Individuals at High Risk to Sexually Reoffend [file sj-pdf-1-sax-10.1177_10790632221139166.pdf]
